# Diagnostic performance of volatile organic compounds analysis and electronic noses for detecting colorectal cancer: a systematic review and meta-analysis

**DOI:** 10.3389/fonc.2024.1397259

**Published:** 2024-05-13

**Authors:** Qiaoling Wang, Yu Fang, Shiyan Tan, Zhuohong Li, Ruyi Zheng, Yifeng Ren, Yifang Jiang, Xiaopeng Huang

**Affiliations:** ^1^TCM Regulating Metabolic Diseases Key Laboratory of Sichuan Province, Hospital of Chengdu University of Traditional Chinese Medicine, Chengdu, Sichuan, China; ^2^Second Department of Oncology, Hospital of Chengdu University of Traditional Chinese Medicine, Chengdu, Sichuan, China

**Keywords:** volatile organic compounds, VOCs, electronic nose, E-nose, colorectal cancer, diagnosis

## Abstract

**Introduction:**

The detection of Volatile Organic Compounds (VOCs) could provide a potential diagnostic modality for the early detection and surveillance of colorectal cancers. However, the overall diagnostic accuracy of the proposed tests remains uncertain.

**Objective:**

This systematic review is to ascertain the diagnostic accuracy of using VOC analysis techniques and electronic noses (e-noses) as noninvasive diagnostic methods for colorectal cancer within the realm of clinical practice.

**Methods:**

A systematic search was undertaken on PubMed, EMBASE, Web of Science, and the Cochrane Library to scrutinize pertinent studies published from their inception to September 1, 2023. Only studies conducted on human subjects were included. Meta-analysis was performed using a bivariate model to obtain summary estimates of sensitivity, specificity, and positive and negative likelihood ratios. The Quality Assessment of Diagnostic Accuracy Studies 2 tool was deployed for quality assessment. The protocol for this systematic review was registered in PROSPERO, and PRISMA guidelines were used for the identification, screening, eligibility, and selection process.

**Results:**

This review encompassed 32 studies, 22 studies for VOC analysis and 9 studies for e-nose, one for both, with a total of 4688 subjects in the analysis. The pooled sensitivity and specificity of VOC analysis for CRC detection were 0.88 (95% CI, 0.83-0.92) and 0.85 (95% CI, 0.78-0.90), respectively. In the case of e-nose, the pooled sensitivity was 0.87 (95% CI, 0.83-0.90), and the pooled specificity was 0.78 (95% CI, 0.62-0.88). The area under the receiver operating characteristic analysis (ROC) curve for VOC analysis and e-noses were 0.93 (95% CI, 0.90-0.95) and 0.90 (95% CI, 0.87-0.92), respectively.

**Conclusion:**

The outcomes of this review substantiate the commendable accuracy of VOC analysis and e-nose technology in detecting CRC. VOC analysis has a higher specificity than e-nose for the diagnosis of CRC and a sensitivity comparable to that of e-nose. However, numerous limitations, including a modest sample size, absence of standardized collection methods, lack of external validation, and a notable risk of bias, were identified. Consequently, there exists an imperative need for expansive, multi-center clinical studies to elucidate the applicability and reproducibility of VOC analysis or e-nose in the noninvasive diagnosis of colorectal cancer.

**Systematic review registration:**

https://www.crd.york.ac.uk/prospero/#recordDetails, identifier CRD42023398465.

## 1 Introduction

Colorectal carcinoma (CRC) stands as a substantial global public health concern, with an estimated 1.93 million new cases and 0.93 million deaths in 2020 (1). CRC is known to develop from precursor lesions, in most cases adenomas, through the adenoma-carcinoma sequence (2) which can be diagnosed earlier through screening even in its early stages. Through standardized early diagnosis and treatment, the 5-year survival rate for early-stage CRC could exceed 90% (1). Fecal immunochemical test (FIT) and colonoscopy screening for colorectal cancer are pivotal tools for early diagnosis of colorectal cancer (3). However, the detection performance of FIT falls short, with a miss detection rate of 9-29% for CRC and 60-75% for advanced CRC (4). FIT-positive patients are recommended to undergo colonoscopy, but colonoscopy is painful, expensive, and invasive, with the risk of complications such as perforation and bleeding. So not all FIT-positive individuals undergo regular colonoscopy follow-up (5, 6). Therefore, there is an urgent need for convenient, non-invasive, reliable, simple, and cost-effective diagnostic methods to enhance early diagnosis and screening of colorectal cancer.

The analysis of Volatile organic compounds (VOCs) has been applied as a novel and promising diagnostic technique for exploration of non-invasive colorectal neoplasia biomarker. VOCs constitute the by-products of biochemical processes within the human body and typically mirror metabolic states (7, 8). Pathological conditions precipitate aberrant metabolic processes, resulting in a marked increase in VOC production (9). Investigations into cancer-related VOCs have explored various matrices, including breath, blood, urine, saliva, and feces (10–13). Many studies have demonstrated that the applicability of VOC analysis could be used in cancer diagnosis (14–20).

The electronic nose (e-nose) emerges as an instrument equipped with a suite of sensors endowed with specificity and an adept pattern recognition system capable of discerning both simple and complex odors (21). As a relatively recent development, the e-nose has become widely accepted for detecting diseases, owing to its portability, expeditious, cost-effective, and user-friendly diagnostic capabilities, rendering it particularly suited for routine clinical applications. Multiple researchers (22–24) have substantiated the commendable diagnostic accuracy of available e-nose technologies across diverse indications. Notably, van Keulen et al. (25) analyzed exhaled breath from patients with CRC and advanced adenomas (AAs), proving that the Aeonose electronic nose can distinguish CRC and AAs from controls. Additionally, de Meij et al. (26) reported an e-nose sensitivity of 0.85 and a specificity of 0.87 in CRC detection.

Our article aims to systematically review published studies on VOC analysis and e-nose technology concerning colorectal cancer (CRC) detection. Furthermore, we aim to compare their diagnostic performance, with the aspiration of offering a valuable reference for the application of diagnostic techniques in CRC diagnosis.

## 2 Methods

### 2.1 Registration

This systematic review has been registered with PROSPERO, under registration number CRD42023398465. The Preferred Reporting Items for Systematic Review and Meta-Analysis (PRISMA) guidelines were adhered to in both the identification and reporting phases of this review (27).

### 2.2 Search strategy

A comprehensive literature search encompassing PubMed, Embase, Cochrane Library, and Web of Science was conducted from inception up to September 1, 2023. This search, void of language or data publication restrictions, utilized keywords such as “Volatile Organic Compounds,” “VOCs,” “electronic nose,” “e-nose,” “Colorectal neoplasms,” and “diagnosis” or “diagnostic” as search strategy terms. A detailed search strategy is provided in the **Supplement**.

### 2.3 Study selection

A total of 192 articles were retrieved. The eligibility of each article was assessed through a meticulous examination of titles and abstracts by two independent reviewers (Y.F. and S.Y.T.). Inclusion criteria were as follows: (1) studies conducted on adult subjects; (2) studies involving colorectal patients; and (3) studies that identified evaluating the diagnostic accuracy of using VOC analysis or e-nose technology. Exclusion criteria encompassed: (1) studies lacking information on the number of cases, controls, sensitivity, and specificity; and (2) studies published as review articles or case reports. Discrepancies between reviewers were resolved through consensus or, if necessary, with the involvement of a third investigator (Q.L.W.). A total of 32 articles met the inclusion criteria and were subsequently included in this systematic review.

### 2.4 Data collection process

The data extraction and tabulation process from the selected studies was undertaken by two reviewers (S.Y.T. and R.Y.Z.). **Tables 1**, **2** summarized basic study characteristics, including authorship, country and year of publication, study type, detection medium, analysis method, sample size, CRC stage, statistical analysis methodology, sampler, sensitivity, specificity, and the area under the curve (AUC), as well as accuracy.

**Table 1:** Basic characteristics and outcomes of VOC studies in the analysis.

Study	year	country	Type of Study	Detection medium	Analysis method	No of CRC patients	No of controls	Stage of CRC	Statistical method	Sampler	Sensitivity, %	Specificity, %	AUC, %	Accuracy, %
Altomare et al. (28)	2013	Italy	case-control	breath	GC-MS	37	41 (healthy)	I/II:19 III/IV:18	PNN	Tedlar bag	86	83	85.2	85
Altomare et al. (29)	2015	Italy	case-control	breath	TD-GC-MS	48	55 (healthy)	I/II:28 III/IV:20	PNN	Tedlar	100	97.72	100	98.75
										bag				
Altomare et al. (30)	2020	Italy	case-control	breath	GC-MS	83	90 (non-cancer)	I/II:38 III/IV:42	LRA	ReCIVA	90	93	97.9	NR
Alustiza et al. (31)	2023	Spain	case-control	feces	TD-GC-MS	24	32(healthy) 24(Adenomas)	I/II:7 III/IV:17	ANOVA	plastic container	83	82	85	NR
Arasaradnam et al. (32)	2014	UK	case-control	urine	FAIMS	83	50 (healthy)	NR	FDA	ATLAS sampler	88	60	NR	NR
Batty et al. (33)	2015	UK	case-control	feces	SIFT-MS	31	31 (healthy)	NR	PLS-DA	Nalophan sampler	72	78	NR	75
Bel’skaya et al. (34)	2020	Russia	case-control	salivary	capillary GS	18	16 (noncancer)	I/II:25 III/IV:38	CRT	NR	92.3	100	NR	NR
Bond et al. (35)	2019	UK	case-control	feces	GC-MS	21	60 (non-cancer)	NR	PLS-DA, LRA	OdoReader box	87.9	84.6	82	NR
Bosch et al. (36)	2020	Netherlands	case-control	feces	GC-IMS	14	227 (healthy)	AA:24	LRA, RF,SVM,NN	NR	100	100	96.1	NR
Boulind et al. (37)	2022	UK	cross-sectional	urine	GC-MS	558 (suspected)	NR	NR	ANN	NR	87.8	88.2	89.6	NR
Cheng et al. (38)	2022	Netherlands	cross-sectional	breath	TD-GC-TOF-MS	30	84 (negative colonoscopy)	AA:138	RF	Tedlar bag	80	70	NR	NR
Depalma et al. (39)	2014	Italy	case-control	breath	TD-GC-MS	15	15 (healthy)	NR	LDA	Tedlar bag	96.5	100	NR	NR
Ishibe et al. (40)	2018	Japan	case-control	feces	GC-MS	30	26 (healthy)	NR	PLS-DA	Tedlar bag	90	57.7	NR	75
Leja et al. (41)	2015	NR	case-control	breath	GC-MS	71	131 (healthy)	NR	NR	NR	85	90	NR	88
Lena et al. (42)	2012	Italy	case-control	breath	TD-GC-MS	34	36 (healthy)	NR	SVM	Tedlar bag	83	88	94.4	80
Markar et al. (43)	2019	UK	case-control	breath	SIFT-MS	50	100 (healthy)	NR	LRA	NR	96	76	NR	NR
McFarlane et al. (44)	2019	UK	case-control	urine	FAIMS	56	82 (non-cancer)	NR	RF	NR	69	69	72	NR
Mozdiak et al. (45)	2019	UK	cross-sectional	urine	FAIMS and GC-IMS	163 (positive FOBT)	NR	NR	SLR, GPC	NR	100	100	98	NR
Politi et al. (7)	2021	Italy	case-control	breath	IMR-MS	52	45 (healthy)	NR	LRA	NR	96	93	NR	NR
Psutka et al. (46)	2017	JAPAN	case-control	urine	FAIMS	139	78 (healthy)	NR	PCA	NR	67.4	82.1	NR	NR
Tyagi et al. (47)	2021	UK	case-control	urine	GC-TOF-MS	58	38 (healthy)	I/II:24 III/IV:34	RF, NN	NR	86	81	93	NR
Widlak et al. (48)	2018	UK	cross-sectional	urine	FAIMS	562 (Completed colonoscopy)	NR	NR	PCA	NR	63	63	NR	NR
Zambrana et al. (49)	2012	Spain	case-control	breath	GC-MS	38	43 (healthy)	NR	NR	NR	87.06	76.85	NR	NR

AA, advanced adenomas; ANN, artificial neural network; CRC, colorectal cancer; CRT, Classification and Regression Tree; FDA, Fisher Discriminant Analysis; FOBT, fecal occult blood testing; GPC, Gaussian process classifier; LDA, linear discriminant analysis; LRA, logistic regression analysis; NR, Not reported; NN, Neural Network; PCA, Principal Component Analysis, PLS-DA, partial least squares discriminant analysis; PNN, Probabilistic Neural Network; RF, Random Forest; SLR, Sparse Logistic Regression, SVM, support vector machine.

**Table 2:** Basic characteristics and outcomes of e-nose studies in the analysis.

source	year	country	Type of Study	Detection medium	E-Nose type	NO of CRC patients	No of controls	Stage of CRC	Statistical method	Sampler	Sensitivity, %	Specificity, %	AUC, %	Accuracy, %
Amal et al. (14)	2015	Latvia	case-control	breath	Prototype: 6 nanomaterial sensors (GNP and SWCNTs)	65	122 (healthy)	AA:22	DFA	NR	85	94	NR	91
Altomare et al. (50)	2016	Italy	case-control	breath	PEN3:10 MOS	15	15 (healthy)	I/II:1 III/IV:14	PNN	Tedlar bag	93.3	10	NR	37.78
de Meij et al. (26)	2014	Netherlands	case-control	feces	Cyranose 320:32 conducting polymer sensors	40	57 (healthy)	AA:60	CDA	BD box	85	87	NR	92
Steenhuis et al. (51)	2020	Netherlands	cross-sectional	breath	Aeonose:3 MOS	62	NR	I/II:25 III/IV:37	ANN	NR	88	75	NR	NR
Tyagi et al. (47)	2021	UK	case-control	urine	PEN3:10 MOS	58	38 (healthy)	I/II:24 III/IV:34	RF, NN	NR	91	55	81	NR
van de Goor et al. (52)	2017	Netherlands	case-control	breath	Aeonose:3 MOS	28	100 (HNSCC)	NR	ANN	NR	79	81	NR	81
van Keulen et al. (25)	2019	Netherlands	cross-sectional	breath	Aeonose:3 MOS	447t (colonoscopy patients)	NR	NR	ANN	NR	95	64	74	84
Westenbrink et al. (53)	2015	UK	case-control	urine	WOLF:13 electro-chemical sensors	39	18 (healthy)	NR	LDA	NR	92	77	NR	NR
Westenbrink et al. (54)	2016	UK	case-control	urine	WOLF:13 electro-chemical sensors	26	23(IBS)	NR	LDA, KNN	sample box	84.1	82.4	NR	NR
Zonta et al. (55)	2020	Italy	cross-sectional	faeces	SCENT A1: 5 semiconductor gas sensors	398 (colonoscopy patients)	NR	NR	SVM	sample box	116	46	22	214

AA, advanced adenomas; ANN, artificial neural network; CDA, canonical discriminant analysis; CRC, colorectal cancer; DFA, discriminant function analysis; HNSCC, head and neck squamous cell carcinoma; IBS, Irritable bowel syndrome patients; KNN, K Nearest Neighbors; LDA, linear discriminant analysis; NR, Not reported; NN, Neural Network; PNN, Probabilistic Neural Network; RF, Random Forest; SVM, support vector machine.

### 2.5 Quality assessment

The Quality Assessment of Diagnostic Studies 2 tool (QUADAS-2) (56) was conducted to assess the quality of the included studies. This evaluation encompassed four domains: patient selection, index test, reference standard, and patient flow and timing. Ratings were assigned as “low risk,” “unclear,” or “high risk”. The assessment was conducted independently by two investigators (Y.F.J. and Z.H.L.), and any disparities were resolved through the involvement of a third investigator (X.P.H). The complete QUADAS-2 version can be found in Supplement.

### 2.6 Statistical analysis

This meta-analysis was performed by a bivariate model to obtain summary estimates of sensitivity, specificity, and positive and negative likelihood ratios. The Deeks funnel plot asymmetry test was employed to discern publication bias (57). A two-sided *P*<0.10 was deemed statistically significant. Statistical heterogeneity was evaluated among pooled studies using I^2^ index. STATA software (version 16 SE; Stata Corporation, College Station, TX, USA) was used to aggregate analysis and the statistical package MIDAS was used for bivariate meta-analysis and summary receiving operate characteristic (SROC) curve calculation with 95% confidence region. Subgroup analyses were performed by Open Meta-Analyst software to explore sources of heterogeneity based on the characteristics of the included articles.

## 3 Results

### 3.1 Study selection

**Figure 1:**
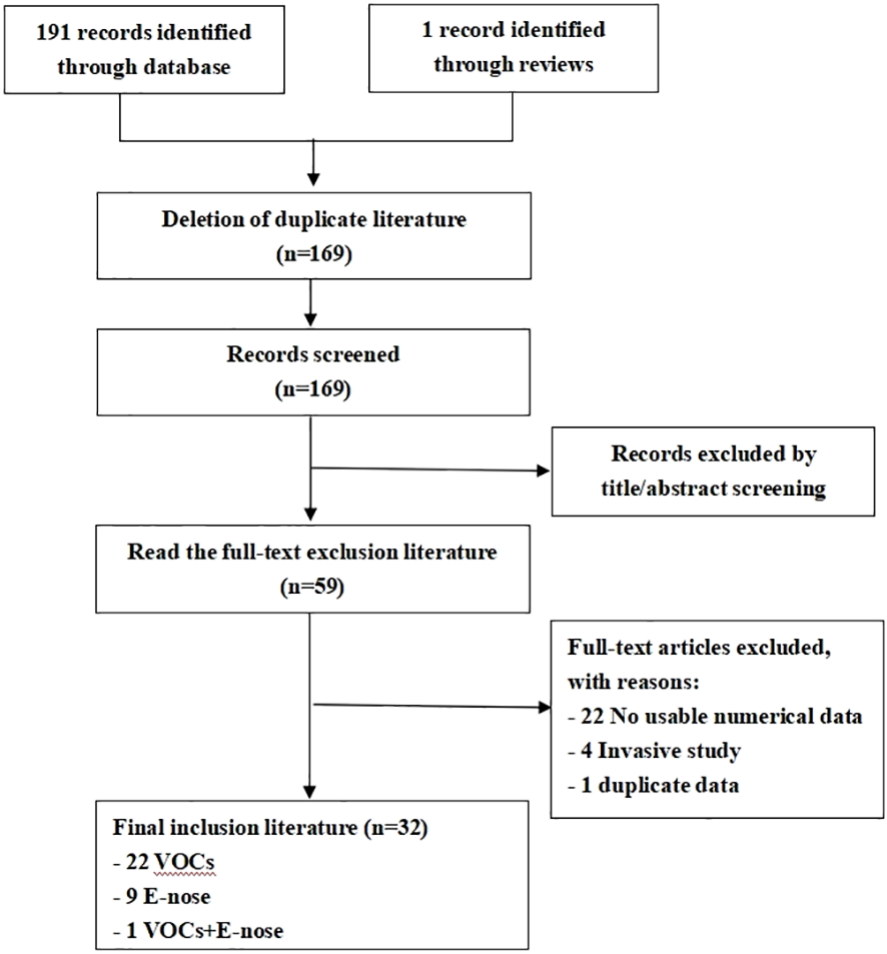
Flow chart of the study selection process.

The literature search strategy yielded an initial pool of 192 articles. Following review, 110 articles were excluded based on title and abstract screening. Subsequently, 59 full-text articles, with a total of 4688 subjects underwent scrutiny against the inclusion criteria. Ultimately, 32 studies fulfilled the inclusion criteria for this review. The selection process of the studies is shown in the PRISMA diagram-**Figure 1**.

### 3.2 Study characteristics

All thirty-two studies included in this review were published in English (7, 25, 26, 28–55, 58). Among them, 22 studies employed VOC analysis for the diagnosis of colorectal cancer (7, 28–46, 48, 49), 9 studies utilized e-nose technology (25, 26, 50–55, 58), and one study used both VOC analysis and e-nose (47). In the VOC studies, 10 studies used breath samples (7, 28–30, 38, 39, 41–43, 49), 6 studies used urine samples (32, 37, 44–46, 48), 5 studies used fecal samples (31, 33, 35, 36, 40), and one study used salivary sample (34). Most studies used MS-based techniques, principally GC-MS (n=7), TD-GC-MS (n=4), FAIM (n=4), and SIFT-MS (n=2). In E-nose studies, 5 studies used breath samples (25, 50–52, 58), two studies used urine samples (53, 54), and two studies used fecal samples (26, 55). One study used both VOC analysis and e-nose technology in testing urine samples (47). The most commonly used e-noses were Aeonose (n=3), PEN3 (n=2), and WOLF (n=2). All studies were prospective, 25 were case-control studies, and 7 employed cross-sectional studies. Logistic regression analysis (LRA) and partial least squares discriminant analysis (PLS-DA) emerged as the most frequently reported analytical methods. Other reported analytical methods encompassed artificial neural network (ANN), support vector machine (SVM), linear discriminant analysis (LDA), random forest (RF), probabilistic neural network (PNN), discriminant function analysis (DFA), and neural network (NN). The majority of studies were conducted in hospital settings, with 29 studies in Europe, two in Asia, and one with an undisclosed location. **Tables 1**, **2** provides an overview of the fundamental characteristics of the studies.

### 3.3 Risk of bias

**Figure 2:**
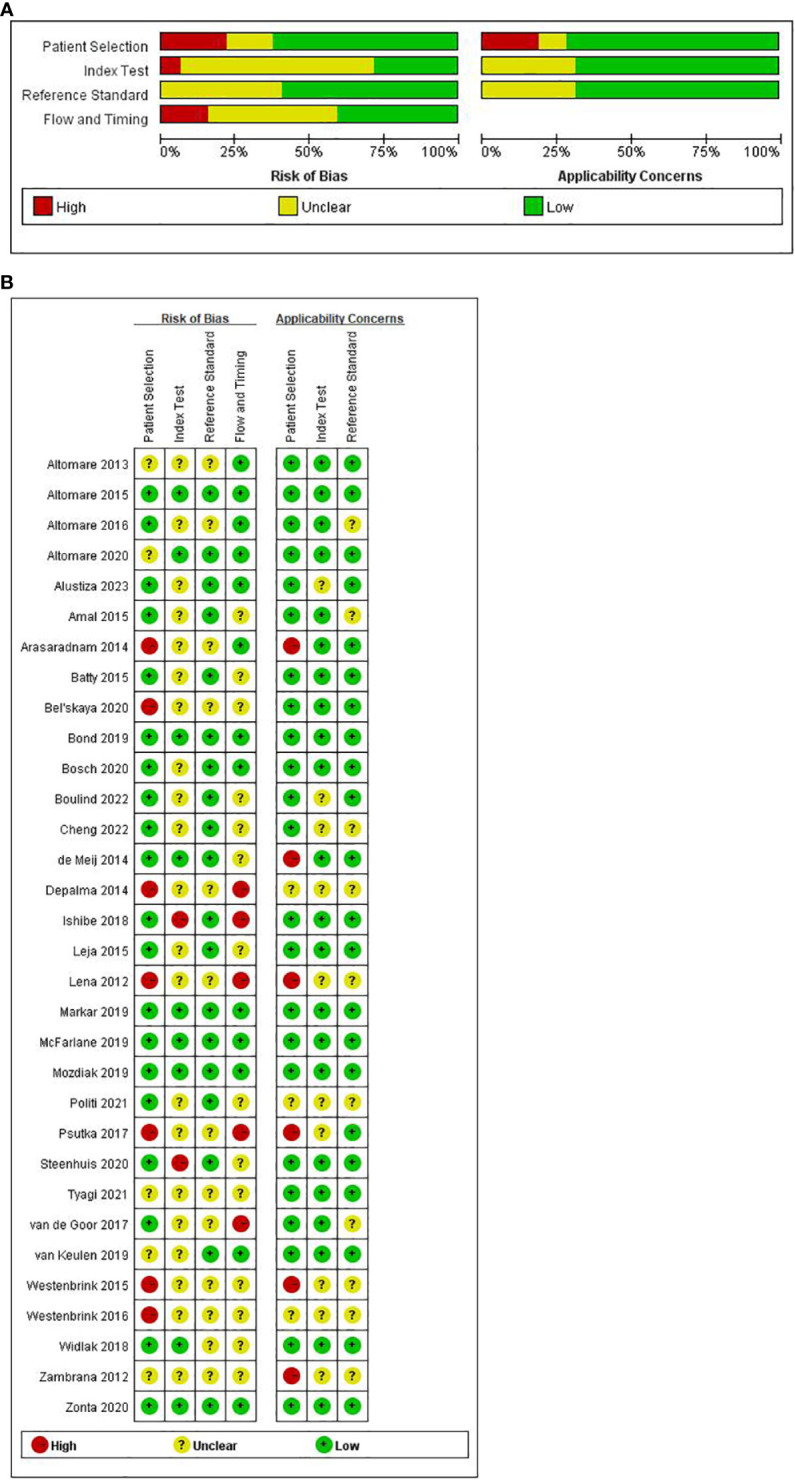
**(A)** Summary and separate outcome of risk of bias and concerns. **(B)** Summary and separate outcome of risk of bias and concerns regarding applicability for included studies using QUADAS-2 tool.

The quality appraisal of all incorporated literature was conducted according to the QUADAS-2 scale through Review Manager 5.4 software. The results of the risk of bias assessment are visually presented in **Figures 2A**, **B**.

In the aggregate, a few studies exhibited a high risk of bias. Concerning ‘patient selection’ seven studies (32, 34, 39, 42, 46, 53, 54) (21.9%) incurred a high risk of bias. The primary contributor to this high risk pertained to the absence of a detailed description of the sampling process and the implementation of a case-control study design. Regarding the ‘index test’ while most studies employed reference diagnostic tests to delineate the definition of a positive test, only nine studies ensured adequate blinding (26, 29, 30, 35, 43–45, 48, 55), leaving 23 studies with an unspecified risk of bias concerning the ‘index test’. Concerning ‘reference standard’, none of the 13 studies (28, 32, 34, 39, 42, 46–50, 52–54) reported the reference standard test. Concerning ‘flow and timing’, five studies (39, 40, 42, 46, 52) faced a high risk of bias. The primary reason for this was that these studies do not account for the time interval between the index test and the reference test.

In evaluating clinical applicability, significant concerns in patient selection arose from the absence of matched patient groups, inadequate patient selection criteria, and applicability of the study design to the research question. Six studies exhibited a high applicability concern for patient selection criteria (26, 32, 42, 46, 49, 53). No high-risk concerns were identified regarding the applicability of the index and reference tests to the research questions.

### 3.4 Diagnostic accuracy

**Figure 3:**
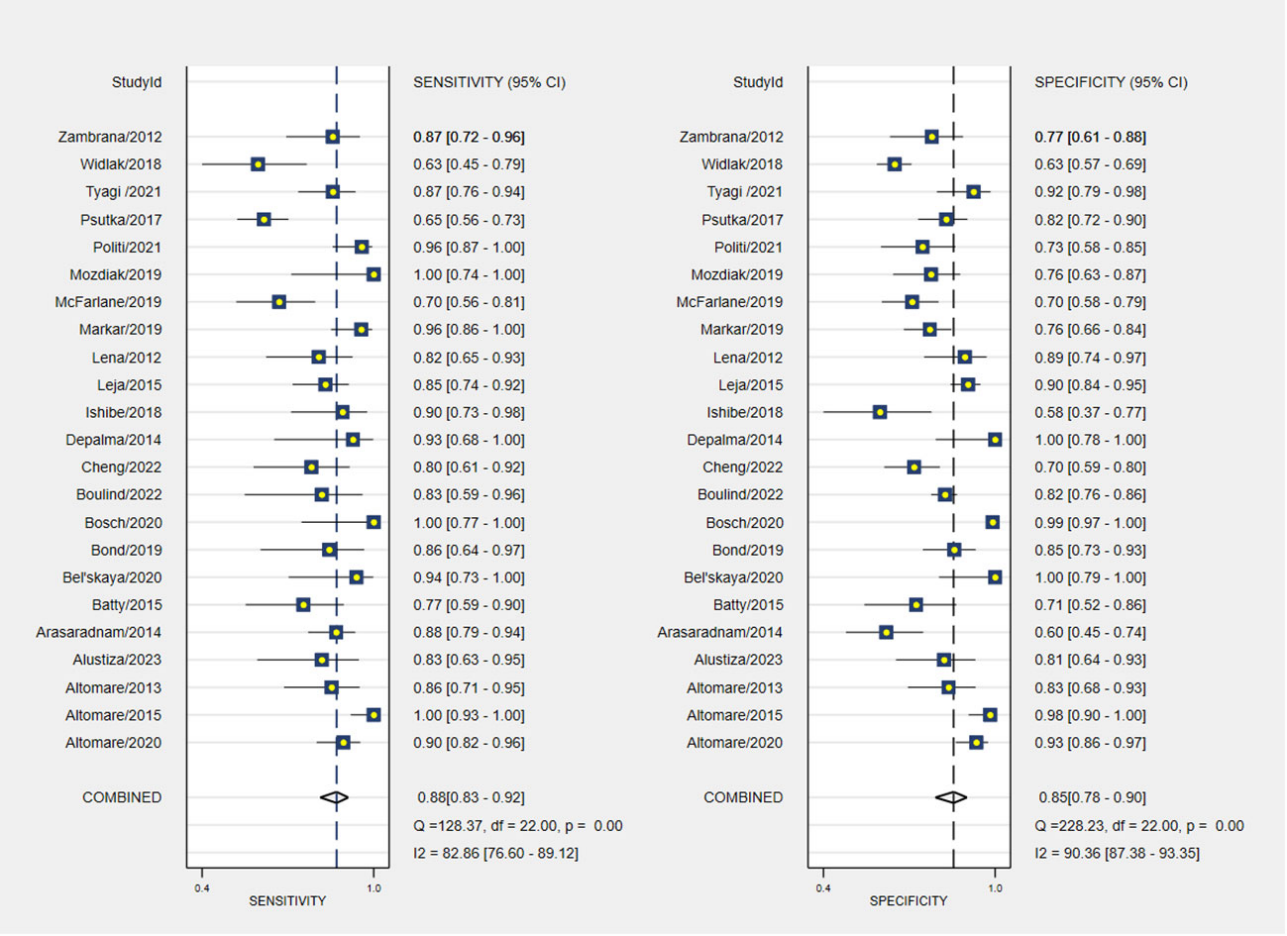
Pooled sensitivity and specificity analyses of VOC studies.

**Figure 4:**
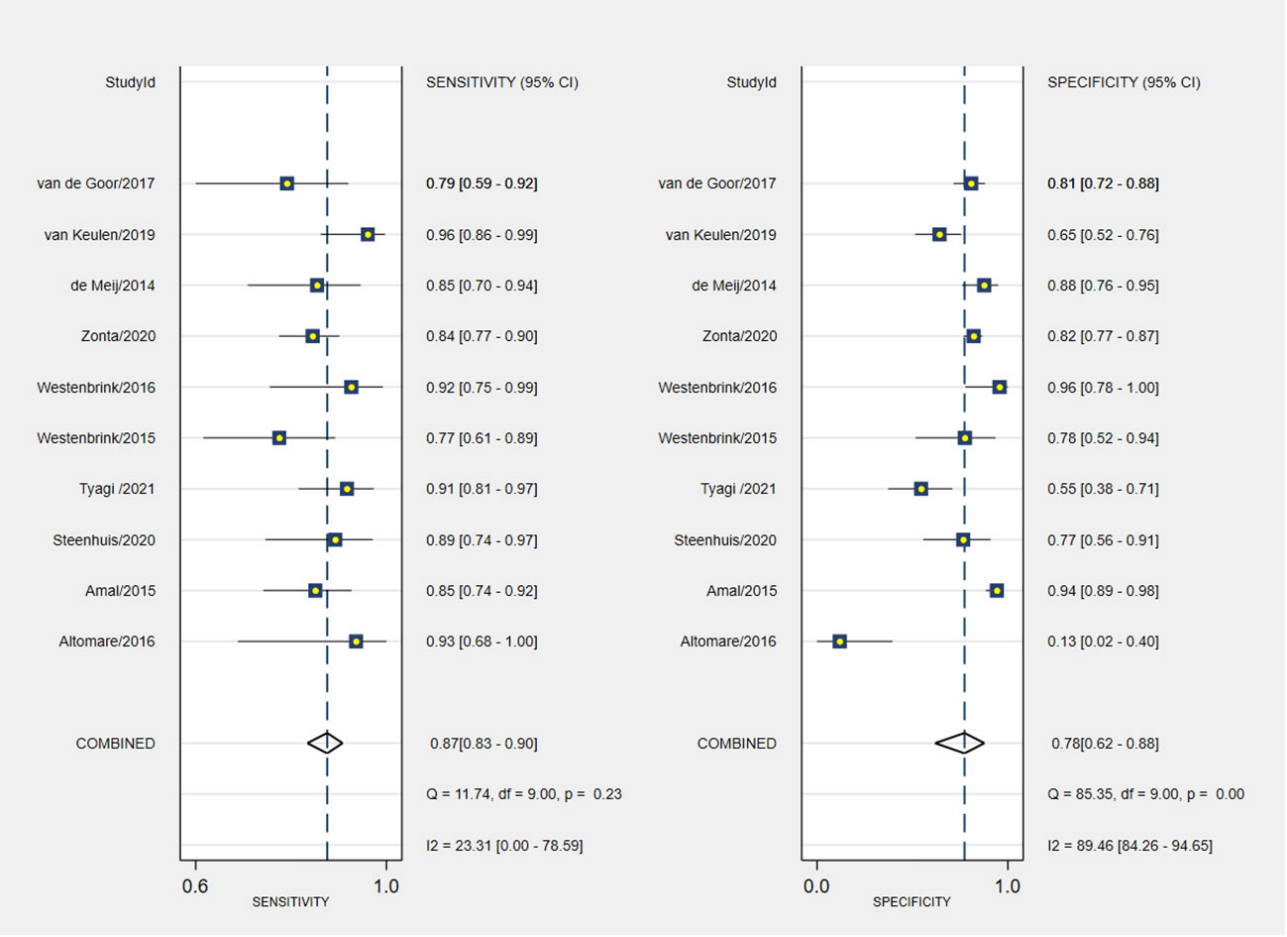
Pooled sensitivity and specificity analyses of e-noses studies.

**Figure 5:**
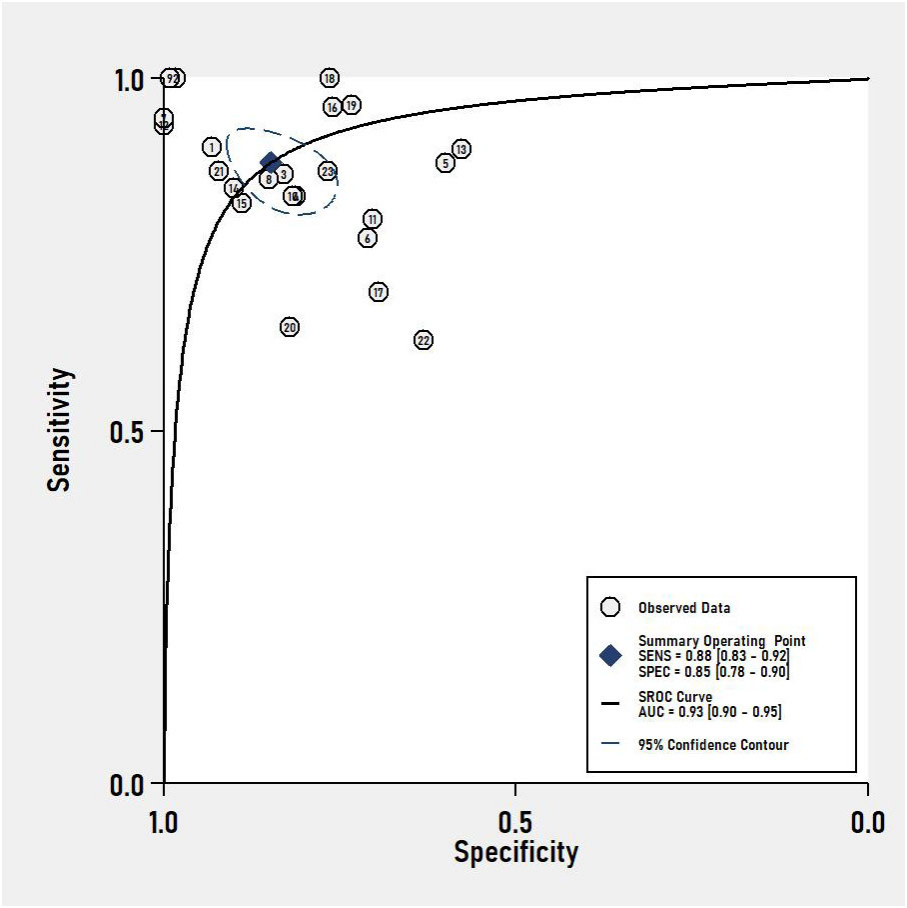
Summary receiver operating characteristic (SROC) curve Analysis of VOC studies.

**Figure 6:**
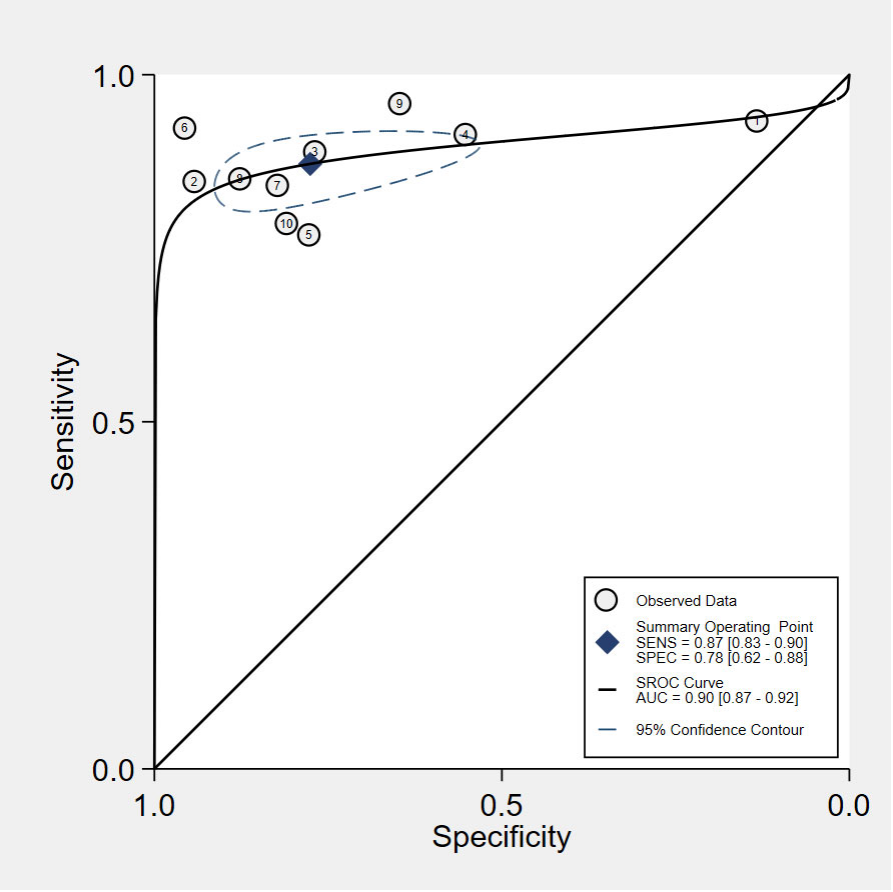
Summary receiver operating characteristic (SROC) curve Analysis of e-noses studies.

The pooled sensitivity and specificity of VOC analysis for detecting CRC were 0.88 (95% CI, 0.83-0.92) and 0.85 (95% CI, 0.78-0.90), respectively (**Figure 3**). Similarly, the pooled sensitivity of the e-nose was 0.87 (95% CI, 0.83-0.90), with a specificity of 0.78 (95% CI, 0.62-0.88) (**Figure 4**). Notably, in VOC studies, the I^2^ index was 82.86% for sensitivity and 90.36% for specificity, while for e-nose studies, it was 23.31% for sensitivity and 89.46% for specificity. Pooled receiver operating characteristic analysis of VOC studies resulted in an area under the curve (AUC) of 0.93 (95% CI, 0.90-0.95) (**Figure 5**). For e-nose studies, the AUC was 0.90 (95% CI, 0.87-0.92) (**Figure 6**). The Positive Likelihood Ratio (PLR), Negative Likelihood Ratio (NLR), and Diagnostic Odds Ratio (DOR) of VOC studies were 5.8 (95% CI, 3.9-8.7), 0.14 (95% CI, 0.09-0.21), and 41 (95% CI, 19-87), respectively. For e-nose studies, the PLR, NLR, and DOR were 3.9 (95% CI, 2.2-6.7), 0.17 (95% CI, 0.13-0.21), and 23 (95% CI, 13-44), respectively.

**Figure 7:**
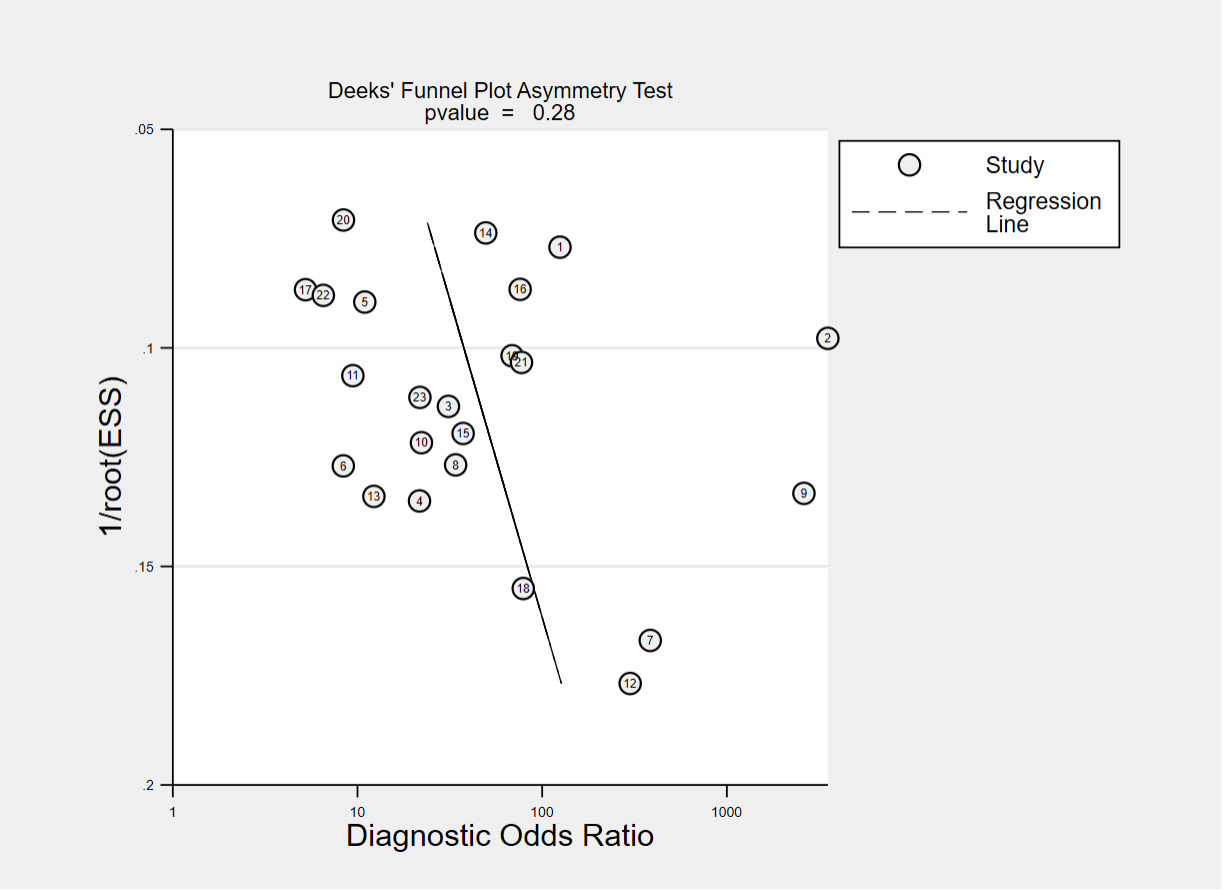
Public bias analysis of all the VOC studies.

**Figure 8:**
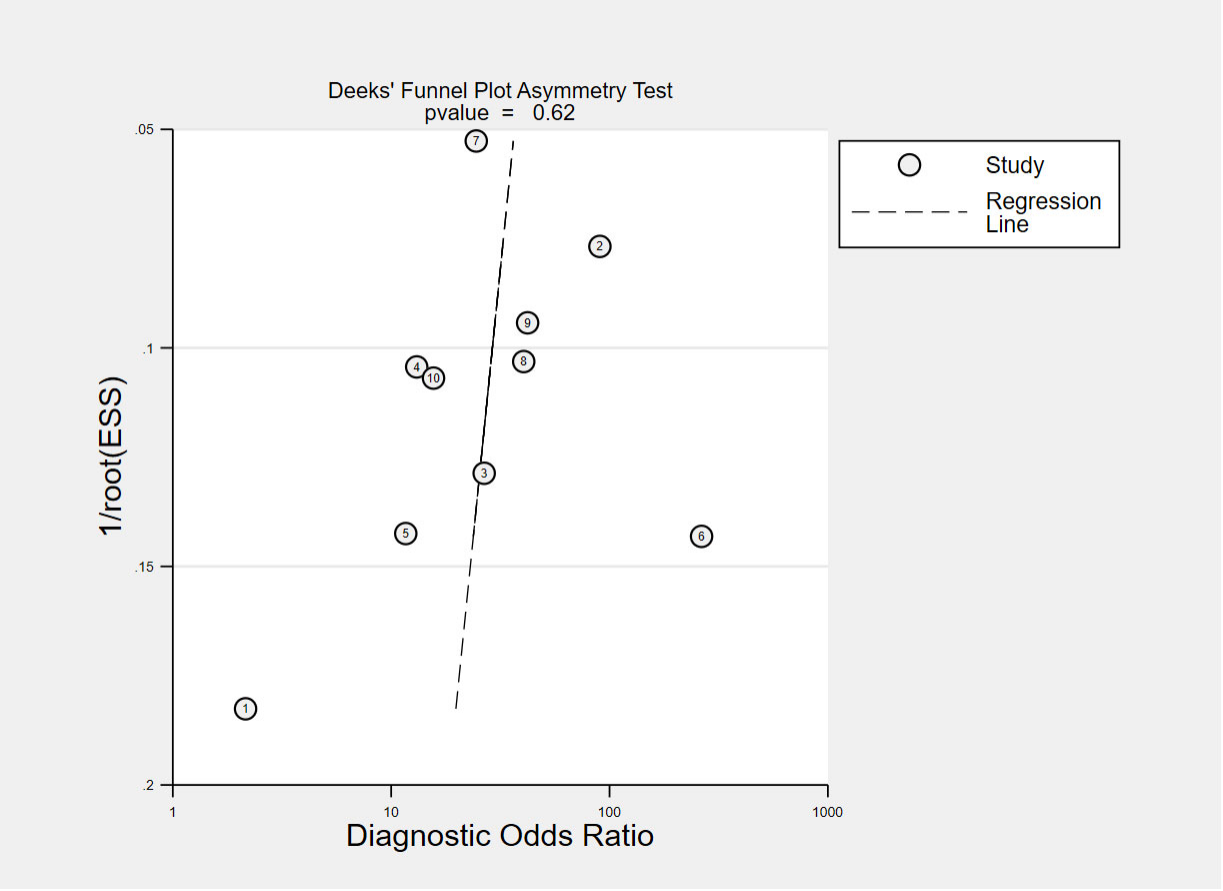
Public bias analysis of all the e-nose studies.

The funnel plots for publication bias are displayed in **Figures 7**, **8**. The Deeks’ regression test for funnel plot asymmetry demonstrated an absence of publication bias among the studies included, with slope coefficients P values of 0.28 and 0.62 for using VOC analysis and e-nose.

### 3.5 Subgroup analysis

We compared the accuracy of different samples of included studies. A separate pooled analysis of breath VOCs studies exhibited good efficacy, with a sensitivity of 0.819 (95% CI, 0.720-0.888) and a specificity of 0.907 (95% CI, 0.876-0.932) (**Table 3**). A Separate pooled analysis of GC-MS, TD-GC-MS, and FAIMS methods, showed a sensitivity of 0.732 (95%CI, 0.519-0.874) and a specificity of 0.919 (95%CI, 0.867-0.952) for GC-MS, and a sensitivity of 0.898 (95% CI, 0.756-0.962) and a specificity of 0.889 (95% CI, 0.783-0.947) for TD-GC-MS, and a sensitivity of 0.635 (95% CI, 0.299-0.877) and a specificity of 0.775 (95% CI, 0.568-0.901) for FAIMS (**Table 3**).

**Table 3:** Subgroup analysis in VOC studies.

Subgroup	Sensitivity (95% CI)	I^2^	Specificity (95% CI)	I^2^
Detects medium				
Breath Samples (n=10)	0.819 (0.720, 0.888)	80.64%	0.907 (0.876, 0.932)	15.58%
Urine Samples (n=7)	0.627(0.365, 0.831)	95.68%	0.862 (0.710, 0.941)	92.09%
Fecal Samples (n=5)	0.730 (0.649, 0.797)	0%	0.905 (0.769, 0.965)	72.52%
The sample analysis method used				
GC-MS (n=7)	0.732 (0.519, 0.874)	0%	0.919 (0.867, 0.952)	60.11%
TD-GC-MS (n=4)	0.898 (0.756, 0.962)	34.83%	0.889 (0.783, 0.947)	30.68%
FAIM (n=4)	0.635 (0.299, 0.877)	3.8%	0.775 (0.568, 0.901)	92.47%
CRC stage				
Advanced adenomas VS. non-cancer control (n=3)	0.824 (0.770, 0.867)	0%	0.908 (0.658, 0.981)	94.03%

For e-nose studies, exhaled breath samples demonstrated a better specificity of 0.911 (95% CI, 0.859-0.945) but a lower sensitivity of 0.708 (95% CI, 0.543-0.833) (**Table 4**). A separate pooled analysis for different types of e-Nose demonstrated that Aeonose could detect colorectal with a sensitivity of 0.682 (95% CI, 0.506-0.817) and a specificity of 0.916 (95% CI, 0.832-0.960). Separate pooled analysis for PEN3 showed a sensitivity of 0.654 (95% CI, 0.401-0.843) and a specificity of 0.791 (95% CI, 0.605-0.903). For WOLF the sensitivity was 0.906 (95%CI, 0.790-0.961) and the specificity was 0.790 (95%CI, 0.359-0.962) (**Table 4**).

**Table 4:** Subgroup analysis in e-nose studies.

Subgroup	Sensitivity (95% CI)	I^2^	Specificity (95% CI)	I^2^
Detects medium				
Breath Samples (n=5)	0.708 (0.543, 0.833)	80.64%	0.911 (0.859, 0.945)	15.58%
Urine Samples (n=3)	0.857 (0.689, 0.942)	95.68%	0.786 (0.563, 0.913)	92.09%
Fecal Samples (n=2)	0.758 (0.631, 0.852)	0%	0.904 (0.864, 0.933)	72.52%
E-Nose type				
Aeonose (n=3)	0.682 (0.506, 0.817)	74.62%	0.916 (0.832, 0.960)	37.95%
PEN3 (n=2)	0.654 (0.401, 0.843)	79.99%	0.791 (0.605, 0.903)	0%
WOLF (n=2)	0.906 (0.790, 0.961)	2.06%	0.790 (0.359, 0.962)	80.97%
CRC stage				
Advanced adenomas VS. non-cancer control (n=3)	0.755 (0.609, 0.859)	55.43%	0.704 (0.628, 0.770)	0%

Additional sensitivity analysis for advanced adenomas demonstrated good accuracy in VOC analysis, with a sensitivity of 0.824 (95% CI, 0.770-0.867) and specificity of 0.908 (95% CI, 0.658-0.981) (**Table 3**). For e-nose studies, the sensitivity and specificity for the detection of advanced adenomas were 0.755 (95% CI, 0.609-0.859) and 0.704 (95% CI, 0.628-0.770), respectively (**Table 4**).

## 4 Discussion

We conducted a systematic review and meta-analysis to evaluate VOC analysis and electronic nose in detecting colorectal cancer, aiming to compare the diagnostic accuracy and clinical application value of these two methods. Pooled analysis of VOC and electronic-nose studies demonstrated high diagnostic accuracy for CRC detection, with a pooled sensitivity of 0.88 and specificity of 0.85 for VOC analysis and a sensitivity of 0.87 and specificity of 0.78 for e-nose studies. The visually assessed SROC curves indicated clinical accuracy, with VOC analysis and e-nose having SROC curves of approximately 0.93 and 0.90, respectively, both close to 1, signifying superior accuracy and diagnostic efficacy in CRC diagnosis. These findings align with prior reviews (22, 59, 60), but the notable heterogeneity between studies and the identified high risk of bias warrant cautious interpretation. The heterogeneity was largely due to the sample media and the analytical methods used.

Subgroup analyses revealed that breath samples in VOC analysis and urine and breath samples in e-nose studies exhibited higher sensitivity or specificity. Breath sampling is easily performed and well-received by patients, and urine samples, boasting high sensitivity and specificity, emerge as valuable alternatives. Recent meta-analysis evaluated the performance of the combined FIT and urinary. The findings revealed that the combined FIT-VOC approach could detect 33% more cases of colorectal cancers (60). Chandrapalan S et al. (61) showed that the combination of FIT and VOC can be a better triage tool, for CRC in patients with lower gastrointestinal symptoms than FIT alone.

Due to the lack of standardization in sample collection, handling, and storage, technical barriers exist in measuring and analyzing various VOC characteristics during sampling, whether it involves alveolar air, urine, or feces. In several studies, exhaled breath was collected into a bag and subsequently analyzed (28, 29, 33, 38–40, 42, 50). The use of bag collection aligns more closely with real-world medical applications. However, this approach may be influenced by several factors, including interference from ambient VOCs, the material used for collection, and the impact of temperature, humidity, and storage time on specimens (62). For breath samples, it is essential to examine them within 6 hours of the collection’s conclusion to ensure test accuracy (63). Therefore, developing methods for the collection, transmission, and handling of breath samples is crucial for the success of this approach. Some studies have indicated that the diagnostic accuracy of fecal and urine VOCs is not significantly affected by storage time (20 months for fecal and 12 months for urine VOCs) (64, 65).

Urine samples are ideal detection medium because they have limited confounding factors compared to breath samples which is influenced by smoking or fecal samples influenced by diet. Further research should standardize the method of collection of such samples and investigate the effects of potential confounding factors.

Among all studies, only six reported on CRC stages, indicating limited generalizability and clinical applicability. Multi-center validation studies of the diagnostic performance of VOCs on early stages of CRC and its precursor lesions (adenomas or not) is warranted, which could reduce the incidence of CRC.

It has been demonstrated that various factors, such as age, gender, smoking, alcohol consumption, coffee intake, and the consumption of stimulating foods like leeks and garlic, as well as comorbidities and medication, may influence the composition of VOCs in exhaled breath (66). However, only a few studies considered confounding or modifying effects, limiting the validity and reliability of the results. Therefore, future studies should account for the impact of such factors on breath prints during the design phase.

Gas chromatography-mass spectrometry (GC-MS), a traditional method for VOC analysis, is a highly standardized technique providing qualitative and quantitative information on exhaled VOCs (67, 68). In this study, TD-GC-MS demonstrated high sensitivity and specificity in detecting colorectal cancer, while GC-MS exhibited improved specificity but suboptimal sensitivity. The use of GC-MS and newer mass spectrometry technology devices remains the gold standard for identifying specific VOCs for analysis. However, GC-MS technology is costly and complex, with long analysis times, and it demands a high level of expertise from operators.

Based on sensors, electronic nose technology serves as a novel analytical method for disease diagnosis, offering the advantages of being cost-effective, user-friendly, portable, sensitive, and responsive. Nevertheless, there are existing shortcomings that require refinement in the application of e-nose in clinical practice. Unlike GC-MS and other techniques, e-nose lacks the precision to measure specific types and composition ratios of components in VOCs (24). It also cannot identify specific pathophysiological pathways or therapeutic targets. Furthermore, as the e-nose relies on arrays of gas sensors to distinguish and identify response spectra of mixtures composed of multiple VOCs, the diverse sensor types with distinct signal responses prevent the integration of results from one e-nose with different devices or sensor types (69). Van der Sar IG (70) recommends the establishment of a comprehensive worldwide shared database encompassing patient characteristics and other pretest probabilities.

Various algorithms and methods were employed to analyze VOCs in this study, with PLA-DA and logistic regression analysis emerging as the most commonly used approaches. However, the majority of studies fail to elucidate the rationale behind selecting a specific machine learning model for analysis, only reporting the highest accuracy value, thereby impacting the reliability of the results. Additionally, studies with small sample sizes may compromise the reported accuracy. Few studies have conducted external validation to affirm the validity and reliability of these findings. Consequently, large, multi-center external validation studies should be conducted in the future to explore the applicability and reproducibility of the results in different study settings and among diverse target populations.

### 4.1 Limitation

This study has certain limitations. Heterogeneity was observed among studies, potentially attributed to variations in sample media and analytical methods. Some studies exhibited a high risk of bias, with seven showing concern regarding patient selection and ten having applicability concerns in one or two domains. Furthermore, the study included fewer investigations employing both VOC analysis and e-nose technology, thus impeding an accurate evaluation of the complementary effects of the two methods. In addition, VOC combined with FIT approach could increase the detection of colorectal cancer. However, there are no prospective studies evaluating the positive effect on VOC-FIT for screening prior to the onset of CRC.

## 5 Conclusion

Based on our meta-analysis, VOC analysis and e-nose technology show promise in the detection of CRC. However, several milestones must be achieved in colorectal cancer detection with these two non-invasive methods before clinical implementation. Firstly, for patients presenting with common non-specific symptoms, which may be an early indication of CRC, an exhaled breath test or a urine test or FIT+VOC could serve as screening tool. Secondly, electronic nose could be utilized in primary care units and community healthcare centers for mass screening of various intestinal diseases due to their portability, ease of use, cost-effectiveness, speed, and independence from specialized technicians. Thirdly, the identification of colorectal cancer-specific VOC biomarkers and combinations of biomarkers for colorectal cancer diagnosis is still necessary. This requires comprehensive metabolomics studies to elucidate the production of endogenous VOCs and the metabolic transformation of exogenous VOCs in colorectal cancer, aiding in the identification of VOC markers for cancer. Finally, large, multi-center external validation trials should be conducted to verify the generalizability and reproducibility of the results in different research settings and at different stages of CRC.

## Data availability statement

The original contributions presented in the study are included in the article/**Supplementary Material**, further inquiries can be directed to the corresponding authors.

## Author contributions

QW: Formal analysis, Funding acquisition, Supervision, Writing – original draft. YF: Investigation, Project administration, Writing – original draft. ST: Methodology, Writing – review & editing. ZL: Data curation, Project administration, Writing – review & editing. RZ: Data curation, Investigation, Writing – review & editing. YR: Methodology, Project administration, Writing – original draft. YJ: Project administration, Software, Writing – original draft. XH: Supervision, Validation, Writing – review & editing.

## References

[1] Sung H Ferlay J Siegel RL Laversanne M Soerjomataram I Jemal A . Global cancer statistics 2020: GLOBOCAN estimates of incidence and mortality worldwide for 36 cancers in 185 countries. CA: Cancer J Clin. (2021) 71:209–49. doi: 10.3322/caac.21660 33538338

[2] Dekker E Tanis PJ Vleugels JLA Kasi PM Wallace MB . Colorectal cancer. Lancet. (2019) 394:1467–80. doi: 10.1016/S0140-6736(19)32319-0 31631858

[3] Shaukat A Levin TR . Current and future colorectal cancer screening strategies. Nat Rev Gastroenterol Hepatol. (2022) 19:521–31. doi: 10.1038/s41575-022-00612-y PMC9063618 35505243

[4] Imperiale TF Gruber RN Stump TE Emmett TW Monahan PO . Performance characteristics of fecal immunochemical tests for colorectal cancer and advanced adenomatous polyps: A systematic review and meta-analysis. Ann Internal Med. (2019) 170:319–29. doi: 10.7326/M18-2390 30802902

[5] Bertels L Lucassen P van Asselt K Dekker E van Weert H Knottnerus B . Motives for non-adherence to colonoscopy advice after a positive colorectal cancer screening test result: a qualitative study. Scandinavian J primary Health Care. (2020) 38:487–98. doi: 10.1080/02813432.2020.1844391 PMC7781896 33185121

[6] Chen H Li N Ren J Feng X Lyu Z Wei L . Participation and yield of a population-based colorectal cancer screening programme in China. Gut. (2019) 68:1450–7. doi: 10.1136/gutjnl-2018-317124 30377193

[7] Politi L Monasta L Rigressi MN Princivalle A Gonfiotti A Camiciottoli G . Discriminant profiles of volatile compounds in the alveolar air of patients with squamous cell lung cancer, lung adenocarcinoma or colon cancer. Molecules. (2021) 26:550. doi: 10.3390/molecules26030550 33494458 PMC7866040

[8] Shirasu M Touhara K . The scent of disease: volatile organic compounds of the human body related to disease and disorder. J Biochem. (2011) 150:257–66. doi: 10.1093/jb/mvr090 21771869

[9] Hakim M Broza YY Barash O Peled N Phillips M Amann A . Volatile organic compounds of lung cancer and possible biochemical pathways. Chem Rev. (2012) 112:5949–66. doi: 10.1021/cr300174a 22991938

[10] Wang C Li P Lian A Sun B Wang X Guo L . Blood volatile compounds as biomarkers for colorectal cancer. Cancer Biol Ther. (2014) 15:200–6. doi: 10.4161/cbt.26723 PMC3928136 24100612

[11] Sethi S Nanda R Chakraborty T . Clinical application of volatile organic compound analysis for detecting infectious diseases. Clin Microbiol Rev. (2013) 26:462–75. doi: 10.1128/CMR.00020-13 PMC3719490 23824368

[12] Monedeiro F Dos Reis RB Peria FM Sares CTG De Martinis BS . Investigation of sweat VOC profiles in assessment of cancer biomarkers using HS-GC-MS. J Breath Res. (2020) 14:026009. doi: 10.1088/1752-7163/ab5b3c 31766027

[13] de Boer NK de Meij TG Oort FA Ben Larbi I Mulder CJ van Bodegraven AA . The scent of colorectal cancer: detection by volatile organic compound analysis. Clin Gastroenterol Hepatol. (2014) 12:1085–9. doi: 10.1016/j.cgh.2014.05.005 24823289

[14] Amal H Shi DY Ionescu R Zhang W Hua QL Pan YY . Assessment of ovarian cancer conditions from exhaled breath. Int J Cancer. (2015) 136:E614–22. doi: 10.1002/ijc.29166 25159530

[15] Peng G Hakim M Broza YY Billan S Abdah-Bortnyak R Kuten A . Detection of lung, breast, colorectal, and prostate cancers from exhaled breath using a single array of nanosensors. Br J Cancer. (2010) 103:542–51. doi: 10.1038/sj.bjc.6605810 PMC2939793 20648015

[16] Barash O Zhang W Halpern JM Hua QL Pan YY Kayal H . Differentiation between genetic mutations of breast cancer by breath volatolomics. Oncotarget. (2015) 6:44864–76. doi: 10.18632/oncotarget.v6i42 PMC4792597 26540569

[17] Corradi M Poli D Banda I Bonini S Mozzoni P Pinelli S . Exhaled breath analysis in suspected cases of non-small-cell lung cancer: a cross-sectional study. J Breath Res. (2015) 9:027101. doi: 10.1088/1752-7155/9/2/027101 25634546

[18] Bouza M Gonzalez-Soto J Pereiro R de Vicente JC Sanz-Medel A . Exhaled breath and oral cavity VOCs as potential biomarkers in oral cancer patients. J Breath Res. (2017) 11:016015. doi: 10.1088/1752-7163/aa5e76 28165332

[19] Guo L Wang C Chi C Wang X Liu S Zhao W . Exhaled breath volatile biomarker analysis for thyroid cancer. Trans Res. (2015) 166:188–95. doi: 10.1016/j.trsl.2015.01.005 25666355

[20] Qin T Liu H Song Q Song G Wang HZ Pan YY . The screening of volatile markers for hepatocellular carcinoma. Cancer epidemiology Biomarkers Prev. (2010) 19:2247–53. doi: 10.1158/1055-9965.EPI-10-0302 20826831

[21] Gardner JW Bartlett PN . A brief history of electronic noses. Sensors Actuators B: Chem. (1994) 18:210–1. doi: 10.1016/0925-4005(94)87085-3

[22] Scheepers M Al-Difaie Z Brandts L Peeters A van Grinsven B Bouvy ND . Diagnostic performance of electronic noses in cancer diagnoses using exhaled breath: A systematic review and meta-analysis. JAMA network Open. (2022) 5:e2219372. doi: 10.1001/jamanetworkopen.2022.19372 35767259 PMC9244610

[23] Licht JC Grasemann H . Potential of the electronic nose for the detection of respiratory diseases with and without infection. Int J Mol Sci. (2020) 21:9416. doi: 10.3390/ijms21249416 33321951 PMC7763696

[24] van Keulen KE Jansen ME Schrauwen RWM Kolkman JJ Siersema PD . The smell of lung disease: a review of the current status of electronic nose technology. Respir Res. (2021) 22:246. doi: 10.1186/s12931-021-01835-4 34535144 PMC8448171

[25] van Keulen KE Jansen ME Schrauwen RWM Kolkman JJ Siersema PD . Volatile organic compounds in breath can serve as a non-invasive diagnostic biomarker for the detection of advanced adenomas and colorectal cancer. Alimentary Pharmacol Ther. (2019) 51:334–46. doi: 10.1111/apt.15622 PMC7003780 31858615

[26] de Meij TG Larbi IB van der Schee MP Lentferink YE Paff T Terhaar Sive Droste JS . Electronic nose can discriminate colorectal carcinoma and advanced adenomas by fecal volatile biomarker analysis: proof of principle study. Int J Cancer. (2014) 134:1132–8. doi: 10.1002/ijc.28446 23959518

[27] Liberati A Altman DG Tetzlaff J Mulrow C Gøtzsche PC Ioannidis JP . The PRISMA statement for reporting systematic reviews and meta-analyses of studies that evaluate healthcare interventions: explanation and elaboration. BMJ (Clinical Res ed). (2009) 339:b2700. doi: 10.1136/bmj.b2700 PMC2714672 19622552

[28] Altomare DF Di Lena M Porcelli F Travaglio E Longobardi F Tutino M . Exhaled volatile organic compounds identify patients with colorectal cancer. Br J Surg. (2013) 100:144–50. doi: 10.1002/bjs.8942 23212621

[29] Altomare DF Picciariello A Rotelli MT De Fazio M Aresta A Zambonin CG . Effects of curative colorectal cancer surgery on exhaled volatile organic compounds and potential implications in clinical follow-up. Ann Surg. (2015) 262:862–7. doi: 10.1097/SLA.0000000000001471 26583677

[30] Altomare DF Porcelli F Picciariello A Pinto M Di Lena M Caputi Iambrenghi O . Chemical signature of colorectal cancer: case–control study for profiling the breath print. BJS Open. (2020) 4:1189–99. doi: 10.1002/bjs5.50354 PMC8444279 32990407

[31] Alustiza M Ripoll L Canals A Murcia O Martínez-Roca A García-Heredia A . A novel non-invasive colorectal cancer diagnostic method: Volatile organic compounds as biomarkers. Clinica Chimica Acta. (2023) 542:1189–99. doi: 10.1016/j.cca.2023.117273 36863694

[32] Arasaradnam RP McFarlane MJ Ryan-Fisher C Westenbrink E Hodges P Thomas MG . Detection of colorectal cancer (CRC) by urinary volatile organic compound analysis. PloS One. (2014) 9. doi: 10.1371/journal.pone.0108750 PMC4182548 25268885

[33] Batty CA Cauchi M Lourenço C Hunter JO Turner C . Use of the analysis of the volatile faecal metabolome in screening for colorectal cancer. PLoS One. (2015) 10. doi: 10.1371/journal.pone.0130301 PMC4472922 26086914

[34] Bel'skaya LV Sarf EA Shalygin SP Postnova TV Kosenok VK . Identification of salivary volatile organic compounds as potential markers of stomach and colorectal cancer: A pilot study. J Oral Biosci. (2020) 62:212–21. doi: 10.1016/j.job.2020.05.002 32474113

[35] Bond A Greenwood R Lewis S Corfe B Sarkar S O'Toole P . Volatile organic compounds emitted from faeces as a biomarker for colorectal cancer. Alimentary Pharmacol Ther. (2019) 49:1005–12. doi: 10.1111/apt.15140 PMC6593415 30828825

[36] Bosch S Bot R Wicaksono A Savelkoul E van der Hulst R Kuijvenhoven J . Early detection and follow-up of colorectal neoplasia based on faecal volatile organic compounds. Colorectal Dis. (2020) 22:1119–29. doi: 10.1111/codi.15009 32040880

[37] Boulind CE Gould O de Lacy Costello B Allison J White P Ewings P . Urinary volatile organic compound testing in fast-track patients with suspected colorectal cancer. Cancers. (2022) 14:2127. doi: 10.3390/cancers14092127 35565258 PMC9099958

[38] Cheng HR van Vorstenbosch RWR Pachen DM Meulen LWT Straathof JWA Dallinga JW . Detecting colorectal adenomas and cancer using volatile organic compounds in exhaled breath: A proof-of-principle study to improve screening. Clin Trans Gastroenterol. (2022) 13. doi: 10.14309/ctg.0000000000000518 PMC10476860 35981245

[39] Depalma N Di Lena M Porcelli F Travaglio E Longobardi F Demarinis Loiotile A . Detection of colorectal polyps by exhaled VOCs. Preliminary data. Techniques Coloproctology. (2014) 18:92–3. doi: 10.1007/s10151-013-1096-6

[40] Ishibe A Ota M Takeshita A Tsuboi H Kizuka S Oka H . Detection of gas components as a novel diagnostic method for colorectal cancer. Ann Gastroenterological Surg. (2018) 2:147–53. doi: 10.1002/ags3.12056 PMC5881347 29863156

[41] Leja M Amal H Funka K Vanags A Kikuste I Sivins A . Nanoarray sensor technology-based volatile marker tests to detect colorectal cancer and colonic adenomas. Gastroenterology. (2015) 148. doi: 10.1016/S0016-5085(15)30059-7

[42] Lena MD Porcelli F Trizio L Giuratrabocchetta S Travaglio E Tutino M . Colorectal cancer screening by breath analysis: A specific pattern of volatile organic compounts (VOCs) can discriminate between patients and healthy controls. Gastroenterology. (2012) 142:S528. doi: 10.1016/S0016-5085(12)62029-0

[43] Markar SR Chin S-T Romano A Wiggins T Antonowicz S Paraskeva P . Breath volatile organic compound profiling of colorectal cancer using selected ion flow-tube mass spectrometry. Ann Surg. (2019) 269:903–10. doi: 10.1097/SLA.0000000000002539 29194085

[44] McFarlane M Millard A Hall H Savage R Constantinidou C Arasaradnam R . Urinary volatile organic compounds and faecal microbiome profiles in colorectal cancer. Colorectal Dis. (2019) 21:1259–69. doi: 10.1111/codi.14739 31282600

[45] Mozdiak E Wicaksono AN Covington JA Arasaradnam RP . Colorectal cancer and adenoma screening using urinary volatile organic compound (VOC) detection: early results from a single-centre bowel screening population (UK BCSP). Techniques Coloproctology. (2019) 23:343–51. doi: 10.1007/s10151-019-01963-6 PMC6536474 30989415

[46] Psutka C Yamada M Matsuda A Yamahatsu K Matsumoto S Kitayama T . Abstract 5303: FAIMS technology in urinary volatile organic compound analysis to detect colorectal cancer. Cancer Res. (2017) 77:5303–. doi: 10.1158/1538-7445.AM2017-5303

[47] Tyagi H Daulton E Bannaga AS Arasaradnam RP Covington JA . Non-invasive detection and staging of colorectal cancer using a portable electronic nose. Sensors. (2021) 21:5440. doi: 10.3390/s21165440 34450881 PMC8398649

[48] Widlak MM Neal M Daulton E Thomas CL Tomkins C Singh B . Risk stratification of symptomatic patients suspected of colorectal cancer using faecal and urinary markers. Colorectal Dis. (2018) 20:O335–O42. doi: 10.1111/codi.14431 30248228

[49] Zambrana Tevar F Herrero A Vidal-de-Miguel G Bailador G Criado E Marquina I . On-line breath analysis of volatile organic compounds as a method for colorectal cancer detection. J Clin Oncol. (2012) 30:1570–. doi: 10.1200/jco.2012.30.15_suppl.1570

[50] Altomare DF Porcelli F Picciariello A Pinto M Di Lena M Caputi Iambrenghi O . The use of the PEN3 e-nose in the screening of colorectal cancer and polyps. Techniques Coloproctology. (2016) 20:405–9yang. doi: 10.1007/s10151-016-1457-z 27000856

[51] Steenhuis EGM Schoenaker IJH de Groot JWB Fiebrich HB de Graaf JC Brohet RM . Feasibility of volatile organic compound in breath analysis in the follow-up of colorectal cancer: A pilot study. Eur J Surg Oncol. (2020) 46:2068–73. doi: 10.1016/j.ejso.2020.07.028 32778485

[52] van de Goor RMGE Leunis N van Hooren MRA Francisca E Masclee A Kremer B . Feasibility of electronic nose technology for discriminating between head and neck, bladder, and colon carcinomas. Eur Arch Oto-Rhino-Laryngology. (2017) 274:1053–60. doi: 10.1007/s00405-016-4320-y PMC5281663 27730323

[53] Westenbrink E Arasaradnam RP O'Connell N Bailey C Nwokolo C Bardhan KD . Development and application of a new electronic nose instrument for the detection of colorectal cancer. Biosensors Bioelectronics. (2015) 67:733–8. doi: 10.1016/j.bios.2014.10.044 25465796

[54] Westenbrink E O’Connell N Bailey C Nwokolo C Bardhan K Arasaradnam R . Detection of colorectal cancer from urinary volatile organic compounds using a new chromatograph/electronic-nose instrument – wolf system. Gut. (2016) 65:A195.2–A6. doi: 10.1136/gutjnl-2016-312388.361

[55] Zonta G Malagù C Gherardi S Giberti A Pezzoli A De Togni A . Clinical validation results of an innovative non-invasive device for colorectal cancer preventive screening through fecal exhalation analysis. Cancers. (2020) 12:1471. doi: 10.3390/cancers12061471 32512911 PMC7352827

[56] Whiting PF Rutjes AW Westwood ME Mallett S Deeks JJ Reitsma JB . QUADAS-2: a revised tool for the quality assessment of diagnostic accuracy studies. Ann Internal Med. (2011) 155:529–36. doi: 10.7326/0003-4819-155-8-201110180-00009 22007046

[57] Deeks JJ Macaskill P Irwig L . The performance of tests of publication bias and other sample size effects in systematic reviews of diagnostic test accuracy was assessed. J Clin Epidemiol. (2005) 58:882–93. doi: 10.1016/j.jclinepi.2005.01.016 16085191

[58] Amal H Leja M Funka K Lasina I Skapars R Sivins A . Breath testing as potential colorectal cancer screening tool. Int J Cancer. (2016) 138:229–36. doi: 10.1002/ijc.29701 26212114

[59] Hanna GB Boshier PR Markar SR Romano A . Accuracy and methodologic challenges of volatile organic compound-based exhaled breath tests for cancer diagnosis: A systematic review and meta-analysis. JAMA Oncol. (2019) 5:e182815. doi: 10.1001/jamaoncol.2018.2815 30128487 PMC6439770

[60] van Liere E van Dijk LJ Bosch S Vermeulen L Heymans MW Burchell GL . Urinary volatile organic compounds for colorectal cancer screening: A systematic review and meta-analysis. Eur J Cancer (Oxford England: 1990). (2023) 186:69–82. doi: 10.1016/j.ejca.2023.03.002 37030079

[61] Chandrapalan S Bosch S Cubiella J Guardiola J Kimani P Mulder C . Systematic review with meta-analysis: volatile organic compound analysis to improve faecal immunochemical testing in the detection of colorectal cancer. Alimentary Pharmacol Ther. (2021) 54:14–23. doi: 10.1111/apt.16405 34004036

[62] Ghimenti S LoMonaco T Bellagambi FG Tabucchi S Onor M Trivella MG . Comparison of sampling bags for the analysis of volatile organic compounds in breath. J Breath Res. (2015) 9:047110. doi: 10.1088/1752-7155/9/4/047110 26654981

[63] Mochalski P King J Unterkofler K Amann A . Stability of selected volatile breath constituents in Tedlar, Kynar and Flexfilm sampling bags. Analyst. (2013) 138:1405–18. doi: 10.1039/c2an36193k PMC4909142 23323261

[64] Esfahani S Sagar NM Kyrou I Mozdiak E O'Connell N Nwokolo C . Variation in gas and volatile compound emissions from human urine as it ages, measured by an electronic nose. Biosensors. (2016) 6:4. doi: 10.3390/bios6010004 26821055 PMC4810396

[65] Chan DK Leggett CL Wang KK . Diagnosing gastrointestinal illnesses using fecal headspace volatile organic compounds. World J Gastroenterol. (2016) 22:1639–49. doi: 10.3748/wjg.v22.i4.1639 PMC4721995 26819529

[66] Krilaviciute A Leja M Kopp-Schneider A Barash O Khatib S Amal H . Associations of diet and lifestyle factors with common volatile organic compounds in exhaled breath of average-risk individuals. J Breath Res. (2019) 13:026006. doi: 10.1088/1752-7163/aaf3dc 30523935

[67] Boots AW Bos LD van der Schee MP van Schooten FJ Sterk PJ . Exhaled molecular fingerprinting in diagnosis and monitoring: validating volatile promises. Trends Mol Med. (2015) 21:633–44. doi: 10.1016/j.molmed.2015.08.001 26432020

[68] Fiehn O . Metabolomics by gas chromatography–mass spectrometry: combined targeted and untargeted profiling. Curr Protoc Mol Biol. (2016) 114:30.4.1–30.4.32. doi: 10.1002/0471142727.mb3004s114 PMC4829120 27038389

[69] Baldini C Billeci L Sansone F Conte R Domenici C Tonacci A . Electronic nose as a novel method for diagnosing cancer: A systematic review. Biosensors. (2020) 10:84. doi: 10.3390/bios10080084 32722438 PMC7459473

[70] van der Sar IG Wijsenbeek MS Moor CC . Exhaled breath analysis in interstitial lung disease. Curr Opin pulmonary Med. (2023) 29:443–50. doi: 10.1097/MCP.0000000000000978 PMC10399937 37405699

